# Treatment Challenges in a Patient With Two Distinct Malignancies and Brain Metastases

**DOI:** 10.7759/cureus.60728

**Published:** 2024-05-20

**Authors:** Rita Banha, Andreia Coutada, Cátia Faustino

**Affiliations:** 1 Medical Oncology, Hospital do Divino Espírito Santo de Ponta Delgada, Ponta Delgada, PRT; 2 Pathology, Portuguese Oncology Institute of Porto/Porto Comprehensive Cancer Centre Raquel Seruca, Porto, PRT; 3 Medical Oncology, Instituto Português de Oncologia do Porto Francisco Gentil, EPE, Porto, PRT

**Keywords:** urological neoplasms, drug interactions, diagnosis, prostate cancer, brain metastasis

## Abstract

Prostate cancer (PC) is one of the leading causes of cancer death among men worldwide. Brain metastases from PC are very rare, often presenting in advanced stages of the disease, and are associated with a poor prognosis. Treatment is complex and may involve surgery or radiotherapy.

We present the case of a 64-year-old male diagnosed with localized prostate adenocarcinoma, initially treated with pelvic radiotherapy associated with long-term hormonal treatment. While on this hormonal treatment, around one year after radical treatment initiation, he developed bilateral pulmonary metastases, histologically proven to be related to PC, defining a state of metastatic castration-resistant PC. He was asymptomatic and therefore treatment with enzalutamide was initiated. A partial response to the lung lesions was obtained and maintained for more than a year, at which time new mediastinal lymph node metastases were identified. An endobronchial ultrasound biopsy revealed metastases from carcinoma with neuroendocrine differentiation, favoring lung small-cell carcinoma. The patient started chemotherapy with carboplatin and etoposide, with a response. Due to the progression of the mediastinal lymph nodes after eight months, the patient had to undergo chemotherapy again, this time in combination with atezolizumab, with once again partial response. Given the possibility of drug interactions, enzalutamide was suspended during both cycles of chemotherapy and successfully reintroduced afterward. Three months after restarting enzalutamide, he began complaining of headaches. Brain imaging revealed a single frontobasal lesion, without evidence of simultaneous extracerebral progression. Considering the epileptogenic potential of enzalutamide, it was again suspended. The patient underwent surgery and histology revealed metastases of prostate adenocarcinoma, a very rare finding. Systemic re-staging after surgery revealed the progression of cerebral and extra-cerebral disease. The patient is currently proposed for treatment with whole brain radiotherapy and chemotherapy with docetaxel.

This case demonstrates the difficulties associated with the diagnosis and treatment of a patient with two distinct neoplasms. Therapy choices were necessarily adjusted because of significant drug interactions. The diagnosis of brain lesions was the last complication, and it proved to be a challenge as it is a rare entity, with optimal management options not being well established.

## Introduction

Prostate cancer (PC) is one of the leading causes of cancer death among men worldwide [1]. Metastatic disease can present in several forms, with the most common sites of metastases being the bone, distant lymph nodes, liver and thorax [2]. Brain metastases from PC are very rare, often presenting in advanced stages of the disease and are associated with a poor prognosis [3,4]. Clinical manifestations of brain metastases from PC vary with the site of the metastatic focus, and include headaches, seizures, and focal neurological deficits [5].

However, the incidence of brain metastases from PC is increasing, possibly due to the combination of different factors, such as better systemic therapies that increase survival and better diagnostic imaging techniques [3,4,6]. Treatment of these situations is complex, and may involve surgery or radiotherapy, together with supportive therapies [5,6]. Therefore, it should always be discussed in a multidisciplinary manner after considering factors such as the patient's condition and the number and size of lesions.

Contrary to what happens in PC, brain metastases are very common in small-cell cancer patients. In fact, small-cell lung cancer is a highly aggressive disease and it is estimated that up to 50-80% of patients will eventually develop brain metastases [7].

We present the case of a patient whose brain metastases diagnosis and treatment were challenged by the presence of two different primary cancers and by the potential side effects of therapy.

## Case presentation

A 64-year-old male with a personal history of acute myocardial infarction, arterial hypertension, dyslipidemia, sleep apnea, depression and smoking and drinking habits was presented. At the end of 2018, he was diagnosed with prostate adenocarcinoma of the left lobe, Gleason 9 (4+5), with perineural permeation, staged as cT3aN1M0 using pelvic magnetic resonance imaging (MRI), prostate-specific membrane antigen (PSMA) PET scan, bone scintigraphy and thoraco-abdomino-pelvic computed tomography (CT). The initial prostate-specific antigen (PSA) was 5.10 ng/mL. In view of the above, he proposed radical treatment, with radiotherapy associated with long-term hormonal treatment (36 months of goserelin). Radiotherapy was administered to the pelvis (50.4 Gy in 28 fractions, 1.8 Gy per fraction) and simultaneous integrated boost to the prostate and seminal vesicle (70 Gy in 28 fractions, 2.5 Gy per fraction) with volumetric modulated arc therapy (VMAT) technique.

Four months after the end of radiotherapy, a complete imaging response in pelvic magnetic resonance and biochemical response was verified. However, abdominal CT revealed solid pulmonary nodules in the right lung base. Thoracic CT confirmed the existence of bilateral, infra and peri-centimetric pulmonary nodules, with suspicious characteristics. A lung biopsy was performed at the beginning of 2020, which revealed findings compatible with lung metastases from previously diagnosed prostate adenocarcinoma. Analytical evaluation showed a slight increase in PSA (0.99 ng/mL) and testosterone levels compatible with castration. At this time, the patient was asymptomatic, and he was proposed for treatment with enzalutamide (160 mg per day), which he started, with good tolerance.

**Figure 1:**
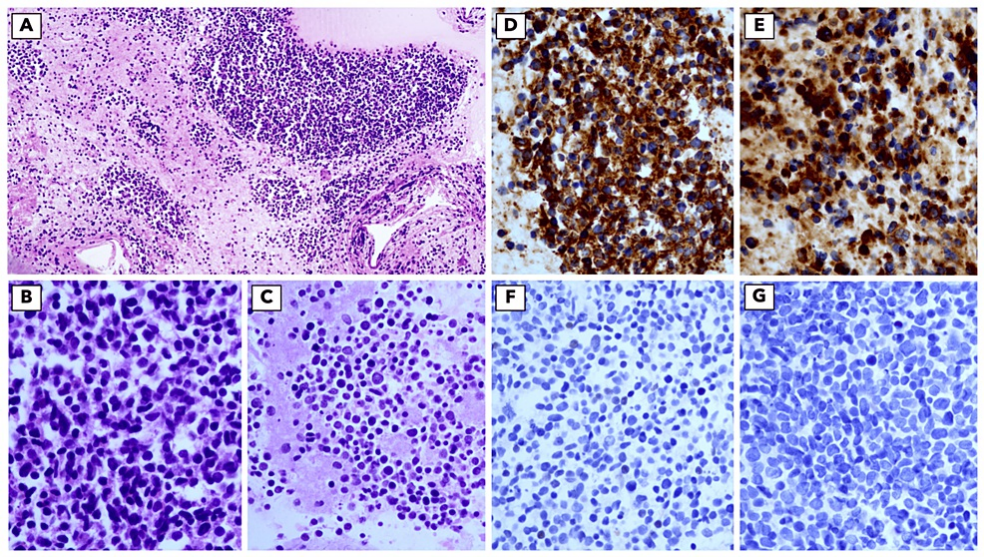
Cytologic features of small-cell neuroendocrine carcinoma A: Cell block showing loosely clustered or dispersed tumour cells (H&E, 10x). B: Small cells with a high N:C ratio showing nuclear moulding and moderate nuclear pleomorphism (H&E, 40x). C: Areas of necrosis (cell-block, H&E, 40x). D: Diffuse positivity for cytokeratin Cam5.2. E: Diffuse positivity for synaptophysin. F: TTF-1 negative. G: PSA negative. N:C ratio: nucleus-to-cytoplasm ratio; TTF-1: thyroid transcription factor 1; PSA: prostate-specific antigen

After three months, a partial response to the pulmonary disease was observed on thoraco-abdomino-pelvic CT. These findings remained stable in subsequent evaluations until 13 months after the start of enzalutamide, when new mediastinal lymph node metastases were reported. PSMA-PET scan revealed possible inflammatory etiology, so a new CT scan was performed after two months, revealing the growth of the mediastinal lesions without progression in previous lung sites or new ones. An endobronchial ultrasound biopsy was performed for clarification, revealing nodal metastases from carcinoma with neuroendocrine differentiation, favoring small cell carcinoma - PSA negative (Figure 1). After careful evaluation of these data by the thoracic and urology oncology teams, a second neoplasm with thoracic origin was assumed, and the patient was proposed for chemotherapy with carboplatin and etoposide, which he started at the end of 2021 (carboplatin dosed at an area under the curve (AUC) of 5 and etoposide 100 mg/m^2^, every 21 days). Given the possibility of drug interactions, enzalutamide was suspended. Hormonal treatment with goserelin was maintained.

After two cycles of chemotherapy, there was a significant volumetric reduction of the mediastinal lesions in the thoraco-abdomino-pelvic CT. He completed six cycles of chemotherapy, without major toxicities. It was possible to resume enzalutamide after the end of chemotherapy, with good tolerance, maintaining stable PSA levels (0.66 ng/mL). He maintained response in subsequent image evaluations for a year, a time when new progression in mediastinal lymph node metastases was verified.

Chemotherapy with platinum and etoposide was again proposed, but this time with a combination of atezolizumab. Once again, he withheld enzalutamide for this purpose. After completion of four cycles of chemotherapy, stable disease was obtained as the best response, thus keeping atezolizumab as maintenance therapy afterwards. Enzalutamide was again resumed, however with an increase in PSA at this time (2.99 ng/mL).

**Figure 2:**
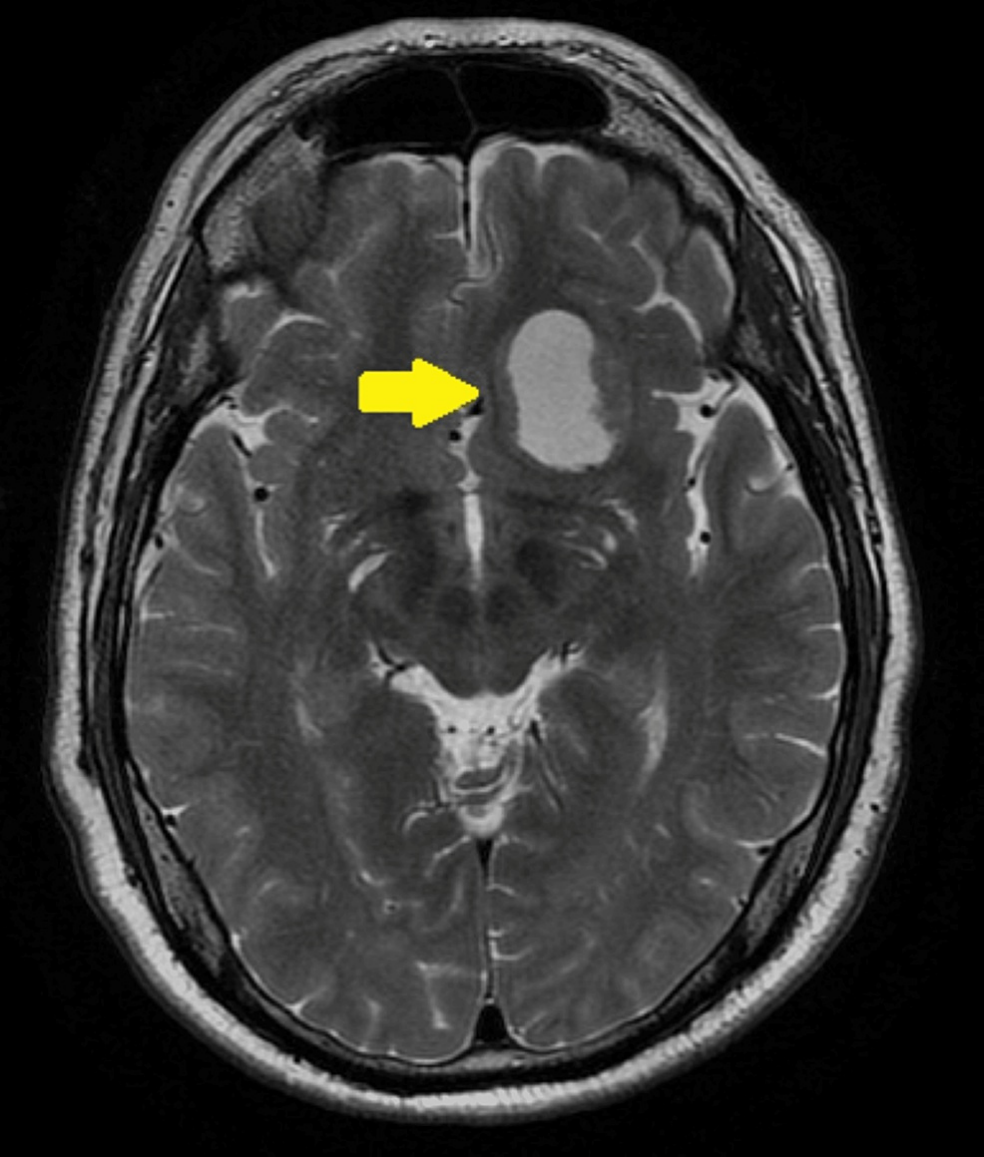
Pre-operative brain magnetic resonance imaging showing an endocranial expansile lesion measuring approximately 33 mm in largest diameter.

Three months after resuming enzalutamide, he began complaints of headaches. Brain CT revealed a single expansive left frontobasal lesion. At this time, both the PSA levels and thoraco-abdomino-pelvic CT remained stable (PSA of 2.91 ng/mL). A brain MRI was performed, which confirmed that it was a single lesion, measuring approximately 33 mm (Figure 2). After discussion in a Multidisciplinary Group with Neurosurgery and Radiation Oncology, surgery was scheduled. Given the epileptogenic potential of enzalutamide, it was again suspended at this time, maintaining goserelin (and atezolizumab).

**Figure 3:**
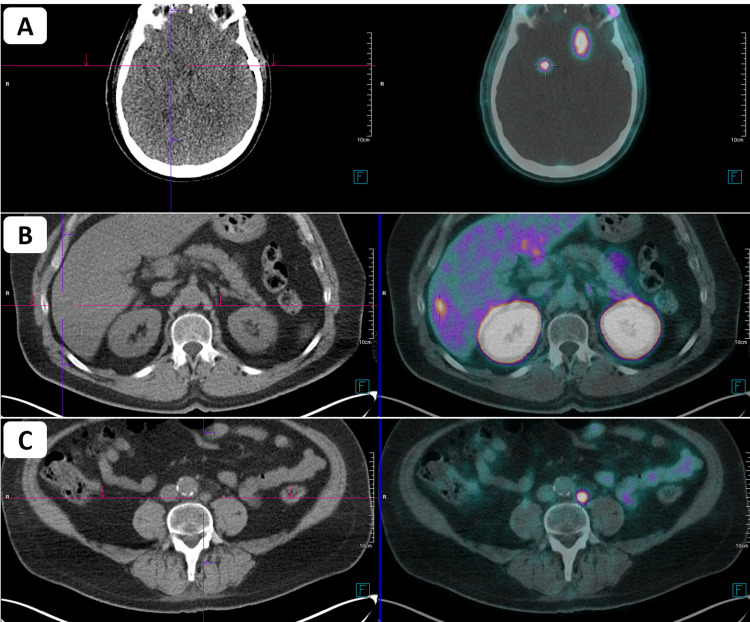
PET-PSMA images demonstrating disease progression after excision of the fronto-basal lesion at the brain, with bilateral cerebral metastases (A), suspicion of liver metastases (B) and lumbo-aortic lymph node metastases (C). PET-PSMA: positron emission tomography-prostate-specific membrane antigen

**Figure 4:**
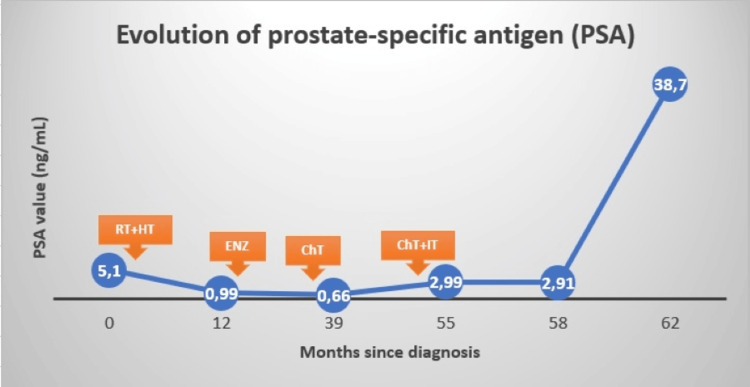
Evolution of prostate-specific antigen (PSA) RT: radiotherapy; HT: hormone therapy; ENZ: enzalutamide; ChT: chemotherapy; IT: immunotherapy

The patient underwent excision of the fronto-basal lesion. Histology revealed metastases of the prostate adenocarcinoma. After the multidisciplinary assessment, it was decided adjuvant stereotactic radiotherapy to the site. However, before adjuvant stereotactic radiotherapy was started, systemic re-staging with PET-PSMA revealed disease progression: brain, with bilateral cerebral metastases, and lumbo-aortic lymph node metastases, both with high PSMA expression (Figure 3). It also revealed suspicion of liver metastases (Figure 3). A new brain MRI was performed, which confirmed bilateral cerebral metastases, with four lesions identified. At this time, the PSA level increased considerably to 38.70 ng/mL (Figure 4). Taking these data into account, the case was again discussed, with a decision to perform whole brain radiotherapy with sparing of the hippocampus and chemotherapy with docetaxel after completing radiotherapy treatments. These treatments are now scheduled to begin.

## Discussion

We present a unique case of a patient with a diagnosis of PC with brain metastases complicated by small-cell carcinoma. Treatment of prostate adenocarcinoma was first challenged at the time of diagnosis of the small cell tumor, as enzalutamide had to be suspended given the possibility of drug interaction with etoposide. In fact, enzalutamide is a strong enzyme inducer of CYP3A4, and it is recommended to avoid concomitant use of enzalutamide with narrow therapeutic index drugs metabolized by CYP3A4 [8]. Maintaining enzalutamide at a lower dose could eventually be discussed, as there is evidence that efficacy is maintained, potentially allowing its concomitant use with other therapies [9]. It was possible to resume enzalutamide after completing chemotherapy, without any complications in this regard.

After that, with the diagnosis of the single brain lesion without evidence of disease progression outside the central nervous system, a new challenge arose. A careful histological evaluation was needed to prove whether the brain lesion was related to the small cell cancer or the prostate adenocarcinoma. Defying the odds, given that metastases to the central nervous system are more common in thoracic than prostate tumors, histology revealed brain metastases of the prostate adenocarcinoma.

In fact, brain metastases from PC are very rare, with reports in the literature estimating an incidence between 0.16% and 0.63% [10]. When brain metastases are present, bone is the most common location for synchronous extracerebral metastases, followed by the liver and lung [3]. Therefore, the absence of bone metastases in this patient is another particular element of this case. The patient underwent successful intervention by Neurosurgery, however, systemic treatment for prostate disease had to be rethought despite the absence of extra-cerebral progression, at the time of cerebral metastase intervention, given that enzalutamide should be used with caution in patients with a history of risk factors of seizures, and, for that very reason, it was assumed to be unsafe to continue to use in this context [11]. Unfortunately, there was evidence of cerebral and extra-cerebral progression on PET-PSMA after surgical intervention.

The prognosis of patients with brain metastases from PC is poor, but can be challenged with the use of chemotherapy, radiotherapy and surgery, together with supportive therapies, such as antiepileptic drugs and corticosteroids [3,6]. Given the rarity of this situation, the most appropriate treatment must be chosen in a multidisciplinary manner, based on multiple factors, namely the number and location of the brain metastases and the control of the systemic disease.

## Conclusions

This unique clinical case shows the difficulties associated with the diagnosis and treatment of a patient with two distinct cancers. The therapeutic path was challenged multiple times by the presence of important drug interactions. Finally, the diagnosis of a single brain metastases related to prostate adenocarcinoma proved to be another difficult situation, given that it is a rare entity, with optimal management options not being well established.
